# Synergistic Probiotic–Postbiotic Therapy Ameliorates Hyperuricemia via Multi-Target Regulation of Purine Metabolism and Gut Microbiota

**DOI:** 10.3390/foods14132213

**Published:** 2025-06-24

**Authors:** Lu Ren, Shiting Liu, Shangshang Wang, Zhenrui Li, Fuping Lu, Xuegang Luo

**Affiliations:** 1 Key Laboratory of Industrial Fermentation Microbiology of the Ministry of Education and Tianjin Key Laboratory of Industrial Microbiology, College of Biotechnology, Tianjin University of Science and Technology, Tianjin 300457, China; 2 National Demonstration Center for Experimental Bioengineering Education, Tianjin University of Science and Technology, Tianjin 300457, China

**Keywords:** hyperuricemia (HUA), probiotics, postbiotics, uric acid metabolism, intestinal flora, synergistic effects

## Abstract

Hyperuricemia (HUA), a metabolic disorder characterized by elevated serum uric acid (UA) levels, often leads to renal and hepatic complications. This study evaluated the synergistic effects of *Pediococcus acidilactici* GQ01, a probiotic strain isolated from naturally fermented wolfberry, in combination with a complex (T) composed of buckwheat-fermented postbiotics, collagen peptide and multiple medicinal food blends in a murine HUA model. The combination therapy (T + GQ01) not only significantly reduced serum UA levels more effectively than T or GQ01 alone but also demonstrated superior inhibition of XOD activity and enhanced ADA activity, both of which are key regulators of purine metabolism. Additionally, T + GQ01 ameliorated kidney injury, as evidenced by reduced serum CRE and BUN levels, and improved liver function, indicated by decreased ALT and AST activities. Histopathological analysis further confirmed the protective effects of T + GQ01 on renal and hepatic tissues. Moreover, T + GQ01 modulated intestinal flora composition, promoted beneficial genera such as *Weissella* and *Bacteroides*, and enhanced the production of SCFAs, particularly propionic and butyric acids, which play critical roles in maintaining intestinal health. These findings suggest that the cocktail-like microecological regulator combining *P. acidilactici* GQ01, buckwheat-fermented postbiotics, collagen peptide and multiple medicinal food blends represents a promising therapeutic strategy for HUA by targeting multiple metabolic pathways, underscoring its potential as a novel intervention for HUA and its complications.

## 1. Introduction

Uric acid (UA) is the end product of purine metabolism, and its production is closely linked to the regulation of purine metabolic pathways [1]. Disruptions in purine metabolism can lead to hyperuricemia (HUA), a clinically prevalent metabolic disorder characterized by elevated serum UA levels [2]. HUA is associated with the deposition of urate crystals in the kidneys and joints, which can result in severe complications such as impaired kidney function, gout, and other metabolic syndromes [3,4]. The growing prevalence of HUA and the limitations of current pharmacological treatments highlight the need for innovative, naturally derived therapeutic strategies [5]. These limitations have spurred interest in developing safer, more effective, and naturally derived alternatives, such as functional foods or dietary supplements, for managing HUA.

In recent years, functional food ingredients characterized by multi-target, multi-active, and low-side-effect properties have attracted attention due to their potential in regulating UA levels. These ingredients mainly include dietary and medicinal food materials, as well as bioactive peptides [6,7]. Examples include sunflower alkaloids, chicory, gardenia, dandelion, corn silk extract, small molecular peptides, and marine fish oligopeptide powder [8]. These natural compounds are known for their ability to modulate metabolic pathways, reduce inflammation, and improve kidney and hepatic function, making them promising candidates for HUA management. Another burgeoning area of research focuses on the role of intestinal flora, probiotics and postbiotics in the regulation of HUA [7,9]. The intestinal–liver axis and intestinal–kidney axis play critical roles in purine metabolism and UA excretion [10]. Dietary interventions that modulate intestinal flora composition have shown potential in improving HUA-related metabolic disorders by enhancing intestinal health and supporting liver and kidney function [11]. Many medicinal food materials exert their therapeutic effects through the modulation of intestinal flora, often mediated by prebiotics or probiotics. *Pediococcus acidilactici* GQ01, a probiotic strain isolated from naturally fermented wolfberry, has demonstrated significant potential in regulating HUA. Previous studies have shown that the live strain of *P. acidilactici* GQ01 can modulate intestinal flora dysbiosis, inhibit the activity of key enzymes involved in UA production, and alleviate liver and kidney damage in HUA mouse models [12]. These findings suggested that *P. acidilactici GQ01* could be developed into a functional food ingredient for HUA management. However, to further optimize its efficacy, it is essential to explore its synergistic effects when combined with other bioactive compounds.

Numerous studies have confirmed that the occurrence and progression of HUA is a complex process involving multiple stages and multiple factors [1]. Consequently, relying solely on a single ingredient or principle to control HUA may not be sufficient. There is a need to explore functional food intervention strategies similar to cocktail therapy. Among numerous potential UA-lowering functional food materials—grape powder, which is rich in polyphenols such as resveratrol—has been demonstrated to inhibit xanthine oxidase, a key enzyme in uric acid production [13]. Goji berry powder, noted for its high antioxidant capacity, may alleviate the oxidative stress associated with HUA [14]. Ginseng powder, containing ginsenosides, exhibits anti-inflammatory effects and may enhance the kidney’s excretion of UA [15]. Jujube powder, poria powder, and sunflower powder contribute additional bioactive compounds that support metabolic homeostasis and reduce inflammation [16]. Tartary buckwheat, rich in flavonoids such as rutin and quercetin, demonstrates the ability to suppress xanthine oxidase activity and decrease uric acid synthesis [17]. Collagen peptides, meanwhile, may support kidney function and enhance uric acid excretion [18]. The probiotic fermentation process further augments the bioavailability and functionality of these compounds, potentially magnifying their therapeutic effects [19]. However, the potential synergistic effects of the aforementioned natural compounds—postbiotics and probiotics in treating HUA remain largely underexplored. In the present study, by combining these ingredients with *P. acidilactici* GQ01, we aimed to develop a multi-targeted, synergistic approach to HUA management. The integration of probiotics, postbiotics, bioactive peptides, and medicinal food materials holds great promise in addressing the complex pathophysiology of HUA. The synergistic interaction between probiotics and functional food complexes may provide a multi-targeted approach to tackle the complex pathophysiology of HUA.

## 2. Materials and Methods

### 2.1. Reagents and Materials

In this study *P. acidilactici* GQ01 and *Lactobacillus casei* IOB-P9 were used (conserved in the General Microbiology Center of China Microbial Strain Conservation and Management Committee, conserved under CGMCC NO.21609 and CGMCC NO.24195). A functional complex (T) composed of buckwheat-fermented postbiotics, collagen peptide and multiple medicinal food blends (grape powder, goji powder, ginseng powder, jujube powder, poria powder and sunflower powder) was prepared by Xi’an Huasheng Bio-Pharmaceutical Co. (Xi’an, China). Potassium oxonate (PO, ≥98% purity) and allopurinol were obtained from Sigma-Aldrich (St. Louis, MO, USA). Yeast extract was sourced from the same supplier. Carboxymethyl cellulose sodium (CMC) was provided by Solarbio Science and Technology Co., Ltd. (Beijing, China). Trizol reagent was acquired from Thermo Fisher Scientific (Waltham, MA, USA). 

### 2.2. Pretreatment

*P. acidilactici* GQ01 strain (2%) was inoculated into liquid MRS (Difco, Tucker, GA, USA) and incubated at 37 °C. After transferring three times, the culture was incubated until the concentration of the bacterial solution was 10^8^ cfu/mL. Sample T, the functional complex composed of buckwheat-fermented postbiotics, collagen peptide and multiple medicinal food blends, was administered 10 mg per rat per day, dissolved in 0.8% saline. The activated *L. casei* IOB-P9 seed culture was inoculated into a solid-state fermentation medium consisting of soybean flour with a material-to-water ratio of 1:1. The inoculation volume was 2% of the dry weight of the medium. Fermentation was carried out at 37 °C for 48 h. The fermented product was then heat-dried at 55 °C until the moisture content was ≤10%, resulting in inactivated *L. casei* IOB-P9 postbiotic powder.

### 2.3. Animal Treatment

A total of 60 male Kunming mice were randomly divided into six groups of 10 mice each according to their body weight, including the negative control group (NC), the HUA model group (M), the positive control allopurinol group (AP), the functional complex group (T), the *P. acidilactici* GQ01 in combination with the functional complex (T + GQ01) and the live *P. acidilactici* GQ01 group (GQ01). The functional complex (T), allopurinol solution, and yeast paste solution required in the experiments were prepared by dissolution using 0.8% saline, and potassium oxonate suspension was prepared from 5% sodium carboxymethylcellulose. In animal experiments, all operations were performed in strict compliance with the principles and requirements of experimental ethics.

After one week of acclimatization, the mice were weighed daily, and interventions were administered based on body weight. Except for the NC group, all mice received 10 g/kg yeast paste by gavage and 300 mg/kg potassium oxonate via intraperitoneal injection daily for modeling. One hour post-modeling, the AP group was treated with 30 mg/kg allopurinol by gavage, the T group received solid-fermented postbiotic peptide, the T + GQ01 group received the same peptide combined with *P. acidilactici* GQ01, and the GQ01 group received *P. acidilactici* GQ01 alone. The NC and M groups were administered an equivalent volume of saline buffer. The experimental cycle lasted 21 days. One hour after the final modeling administration, blood was collected via eyeball removal, and the mice were euthanized by cervical dislocation. Liver, kidney, small intestine, colon contents, and cecum contents were collected and stored at −80 °C for further analysis. The experimental protocol included pre-terminal body weight quantification, followed by post-mortem organometric evaluation. Liver and kidney mass determinations were conducted, enabling the calculation of standardized organ-to-body weight ratios through established normalization procedures [12].

### 2.4. Serum Biochemical Analysis

Blood samples were allowed to stand for 30 min, and then centrifuged at 3000 rpm for 15 min. The supernatant was stored at 4 °C for the measurement of UA, blood urea nitrogen (BUN), creatinine (CRE), glutamate aminotransferase (ALT), aspartate aminotransferase (AST), adenosine deaminase (ADA) and xanthine oxidase (XOD) in the serum. These parameters were measured using commercial kits (Nanjing Jiancheng Chemical Industrial Co., Ltd., Nanjing, China) according to the manuals. Moreover, the measurements of each parameter were carried out in triplicate.

### 2.5. Histopathological Analysis

Hepatic and renal specimens preserved in 4% paraformaldehyde solution underwent sequential processing, including thorough rinsing, fixation in 10% neutral buffered formalin, and subsequent paraffin embedding. Following deparaffinization and rehydration, the samples underwent staining with hematoxylin and eosin (H&E, Tianjin, China). The slides were examined using an orthogonal fluorescence microscope (Olympus BX53, Tokyo, Japan) under blinded conditions. For each group, a minimum of three samples per section were randomly chosen and analyzed at 200× magnification. Images were analyzed using ImageJ software (V1.8.0.112., Rawak Software Inc., Stuttgart, Germany).

### 2.6. Intestinal Analysis by 16S rRNA Gene Sequencing

Cecal contents from mice were collected and sent to Shanghai Major Bio Ltd. (Shanghai, China) for 16S rDNA sequencing analysis. Intestinal flora DNA was extracted using a DNA extraction kit, and the V3 + V4 region of 16S rDNA was amplified by PCR using specific primers 338F (ACTCCTACGGGGAGGCAGCAG) and 806R (GGACTACHVGGGGTWTCTAAT). The PCR products were detected by agarose gel electrophoresis and cut and recovered for purification. The purified PCR products were subjected to secondary PCR, and at the same time connected to the Hiseq2500 PE250 sequencing connector, and sequencing libraries were constructed for sequencing. Finally, clustering and species classification analyses were performed based on the effective sequencing results in terms of Operational Taxonomic Units (OTUs), α-diversity analysis, β-diversity analysis, Hierarchical clustering (Hierarchical clustering), Principal Coordinate Analysis (PCoA), and species difference analysis. Classification and abundance visualization were performed using the Greengene database (Version gg_13_5), Krona software package (version 2.8), etc.

### 2.7. Quantification of Short-Chain Fatty Acids via Gas Chromatographic Analysis

The short-chain fatty acids (SCFAs) in mouse colon contents were analyzed by gas chromatography (GC). Following centrifugation at 4 °C and 4000× *g* for 10 min, the supernatant was extracted. SCFAs were quantified using an Agilent 7890A gas chromatograph equipped with an Agilent 5975C inert XL EI/CI mass spectrometer (MSD) and an HP-5MS capillary column (30 m × 0.25 mm × 0.25 μm, 5% phenyl–95% methylpolysiloxane coating, Agilent, Santa Clara, CA, USA). Helium was utilized as the carrier gas at a constant flow rate of 1 mL/min. The initial column temperature was set at 90 °C, and the temperature was increased to 200 °C in 10 °C steps for 10 min; the pre-sample inlet pressure was 17.732 psi, and the sample was injected into the sample by shunt with a shunt ratio of 1:1, and the initial column temperature was set at 90 °C and kept for 6 min, then the temperature was increased to 200 °C at a rate of 10 °C/min, and kept for 6 min. The massing scan was full wave. A standard curve was made by the external standard method and the concentration of SCFAs (μmol/g sample) was calculated according to the standard curve. The measurements were carried out in triplicate.

### 2.8. Statistical Analysis

Partial least squares discriminant analysis (PLS-DA) including score plot, loading plot, and variable importance in projection (VIP) values, along with the heatmap and correlation analysis, was performed by MetaboAnalyst 5.0 using the online server. Correlation analyses were assessed by Spearman’s rank correlation. Significant differences were analyzed by Student’s *t* test or Kruskal–Wallis test. GraphPad Prism 8.0 was used for data processing and statistical analysis, and One-Way ANOVA was chosen to analyze the differences between groups if the data conformed to normal distribution; if the data did not conform to normal distribution, *T*-test was used for analysis. For the presentation of results, they are usually presented in Mean ± SD format. A *p*-value below 0.05 was deemed statistically significant, reflecting a meaningful difference between groups.

## 3. Results and Discussion

### 3.1. P. acidilactici GQ01, Buckwheat-Fermented Postbiotics, Collagen Peptide and Multiple Medicinal Food Blends Can Synergistically Improve HUA

**Figure 1:**
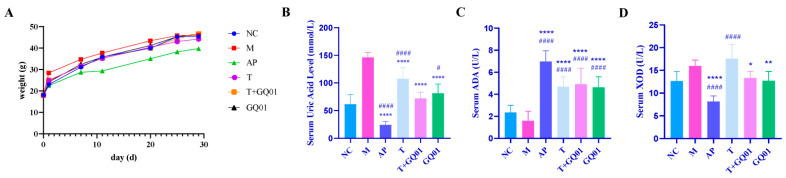
Regulation of body weight, uric acid, and metabolic enzymes by T, T + GQ01, and GQ01 in HUA mice. (**A**) Weight change curve; (**B**) Serum UA level; (**C**) Serum ADA viability; (**D**) Serum XOD viability. Data are presented as mean ± SD (*n* = 10). * or ^#^ *p* < 0.05, ** *p* < 0.01, **** or ^####^ *p* < 0.0001 (^#^ Contrasted with the NC group and * was contrasted with M Group).

Figure 1A shows that high-purine diet modeling increased the body weight in all mice, with the M group showing the most significant gain. In contrast, the AP group exhibited weight loss after 3 days of drug administration, becoming the lowest among all groups. The T, T + GQ01, and GQ01 treatment groups showed higher weight gain than the NC group. After 25 days, the M group’s weight growth rate stabilized, approaching that of the NC group. These results demonstrate that a high-purine diet induces weight gain, while allopurinol treatment for HUA leads to weight loss, consistent with its known adverse effects.

XOD, a key rate-limiting enzyme in purine metabolism, can convert hypoxanthine to xanthine in the liver, and then transform xanthine to UA [20]. ADA, playing a key role in purine nucleoside catabolism, catalyzes the degradation of adenine nucleosides into hypoxanthine nucleosides. Subsequently, nucleoside phosphorylase acts on hypoxanthine nucleosides to produce hypoxanthine, which is ultimately oxidized into the metabolite UA [21]. Figure 1B showed that compared with group M, group AP, group T, group T + GQ01, and group GQ01 all significantly reduced the elevation of uric acid caused by modeling (*p* < 0.001), in which the UA lowering effect of group T + GQ01 was better than that of group T and group GQ01, which suggested that the synergistic effect of these two tested samples was better than the single effect. Figure 1C showed that the ADA enzyme activity was significantly elevated in the AP, T, T + GQ01 and GQ01 groups compared with the NC group (*p* < 0.001), in which the highest ADA enzyme activity was found in the AP group, followed by the T + GQ01 group, indicating that the synergistic effect of the T + GQ01 group had a higher ADA enzyme activity than that of the single-action T and GQ01 groups. As shown in Figure 1D, the XOD enzyme activity in group M was increased through the modeling process. Allopurinol, a xanthine oxidase inhibitor, significantly reduced the XOD enzyme activity in group AP compared to group M (*p* < 0.001). Both group T + GQ01 and group GQ01 also led to a decrease in XOD enzyme activity (*p* < 0.05). However, group T showed an elevation in XOD enzyme activity (*p* < 0.001), and this elevation was significantly greater compared to the NC group (*p* < 0.001), suggesting its therapeutic effect may operate through alternative mechanisms such as enhanced renal urate excretion or modulation of other purine metabolic pathways, rather than direct XOD inhibition. These findings indicated that T + GQ01 could effectively inhibit XOD enzyme activity, and its inhibitory effect was superior to that of single-component treatments. The above results suggested that T, GQ01, and T + GQ01 were all capable of effectively reducing serum UA levels in HUA mice. Notably, the combined treatment of T and GQ01 demonstrated a more pronounced effect compared to either T or GQ01 alone.

### 3.2. P. acidilactici GQ01, Buckwheat-Fermented Postbiotics, Collagen Peptide and Multiple Medicinal Food Blends Synergistically Alleviate Kidney Injury in HUA Mice

**Figure 2:**
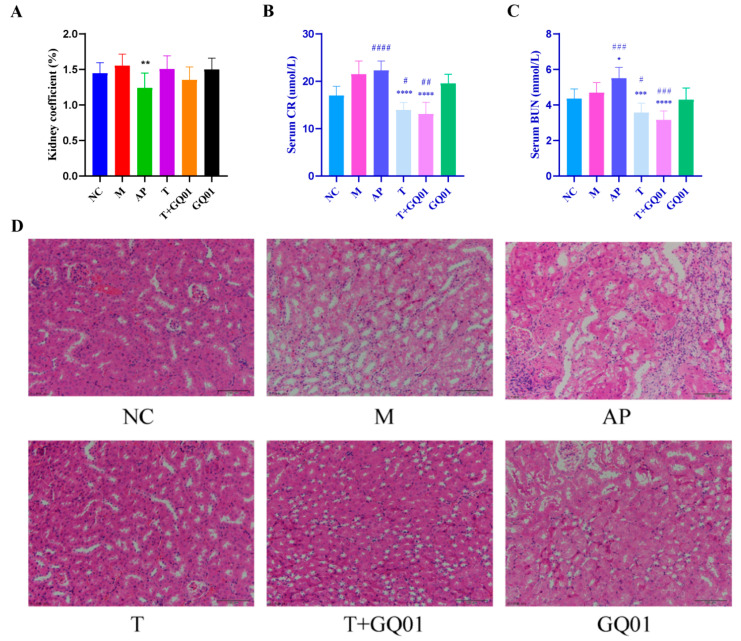
Effects of T, T + GQ01 and GQ01 on kidney injury in HUA model mice. (**A**) Kidney index; (**B**) Serum CR level; (**C**) Serum BUN level; and (**D**) Representative micrographs of H&E-stained mouse kidney tissue sections (200×). * or ^#^ *p* < 0.05, ** or ^##^ *p* < 0.01, *** or ^###^ *p* < 0.001, **** or ^####^ *p* < 0.0001 (^#^ Contrasted with the NC group and * was contrasted with M Group).

As shown in Figure 2A, when compared to the NC group, the kidney coefficient of the mice in the M group exhibited an upward trend; however, this difference was not statistically significant. In contrast, the kidney coefficient of the mice in the AP group was significantly lower (*p* < 0.01), indicating that allopurinol caused kidney injury, which was in line with the observation during autopsy that the kidneys in the AP group were whitened and shrunken. Furthermore, the kidney coefficients in all the remaining experimental groups did not exhibit any statistically significant variations. The kidney is an important place for removing UA, and when human blood uric acid is at a very high level, excess urate crystals will be deposited in the kidney tubules and kidney interstitium, and the obstructing uric acid can activate localized chronic inflammation, immune injury, and microvascular pathology in the kidney, leading to the development of chronic kidney disease [22]. Blood urea nitrogen (BUN) and creatinine (CRE) are two important indicators for evaluating kidney impairment in clinical practice, and abnormal glomerular filtration function leads to abnormal elevation of BUN and CRE [23]. As shown in Figure 2B, serum creatinine was elevated in group M and significantly elevated in group AP compared with group NC (*p* < 0.001); serum creatinine was reduced in groups T, T + GQ01 and GQ01, with a significant reduction in group T + GQ01 (*p* < 0.01); and serum creatinine was significantly reduced in groups T and T + GQ01 compared with group M (*p* < 0.001). Figure 2C showed that serum urea nitrogen was significantly higher (*p* < 0.001) in the AP group and significantly lower (*p* < 0.01) in the T and T + GQ01 groups compared with the NC group; significantly higher (*p* < 0.001) in the AP group, and significantly lower (*p* < 0.001) in the T and T + GQ01 groups compared with the M group. These results indicated that serum BUN and CRE are elevated in HUA mice, and allopurinol also causes kidney injury triggered by elevated serum BUN and CRE, and the interventions of T, T + GQ01, and GQ01 can alleviate this abnormality. Combined with the H&E staining results of Figure 2D, it can be seen that glomeruli of the mice in NC group were intact in structure, with kidney tubules clearly visible, and the kidney cells arranged in a relatively neat manner; in the mice in M group, the kidney tissues of the nuclei were aggregated, and interstitial inflammation appeared in kidney tissue. In group M mice, the nuclei of kidney tissue were aggregated, and there was infiltration of kidney interstitial inflammatory cells, and the distal kidney tubules were dilated, the lumen was enlarged, and there was a tendency of vacuolization of kidney tubular epithelial cells. Compared with group M, the cell size of group T and group T + GQ01 was more uniform, in which the vacuolated cells of group T + GQ01 were significantly reduced, and the kidney tissue structure was basically normalized, while the cells of group AP were widely deformed. These findings suggested that HUA formed through a high-purine diet may lead to kidney injury, and the T group, T + GQ01 group and GQO1 group could improve the kidney tissue and cell morphology to varying degrees and alleviate kidney injury. In addition, severe vacuolization of kidney tubular epithelial cells in allopurinol-treated mice demonstrated that current drugs for alleviating HUA and gout have the function of impairing the body’s metabolism, which is consistent with what the literature reports [24]. Relative to the NC group, the M and AP groups displayed glomerular atrophy, structural deformation, significant tubular lumen dilation, and mild renal edema. Notably, allopurinol administration worsened renal edema and tubular damage, aligning with its documented nephrotoxic properties.

### 3.3. P. acidilactici GQ01, Buckwheat-Fermented Postbiotics, Collagen Peptide and Multiple Medicinal Food Blends Synergistically Alleviate Liver Injury in HUA Mice

**Figure 3:**
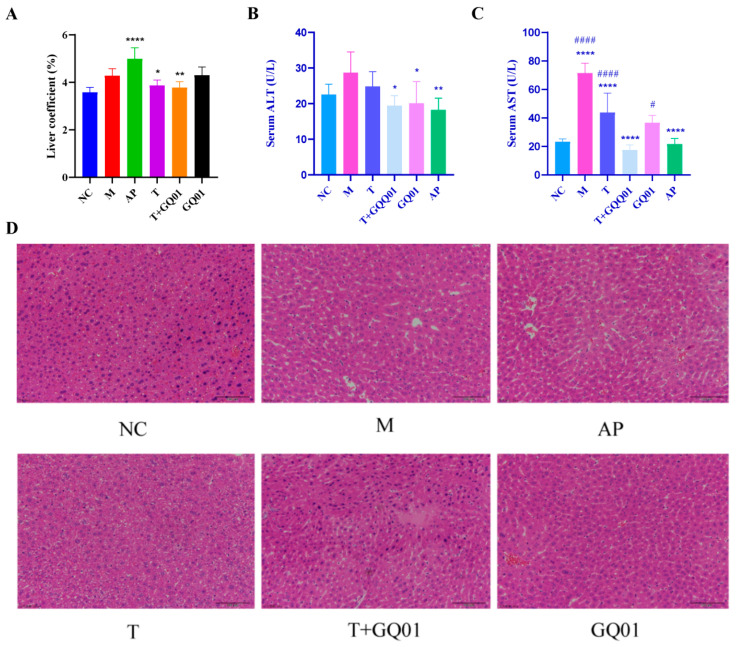
Effects of T, T + GQ01 and GQ01 on liver injury in HUA model mice. (**A**) Liver index; (**B**) Serum ALT level; (**C**) Serum AST level; and (**D**) Representative micrographs of H&E-stained mouse liver tissue sections (200×). * or ^#^ *p* < 0.05, ** *p* < 0.01, **** or ^####^ *p* < 0.0001 (^#^ Contrasted with the NC group and * was contrasted with M Group).

As can be seen in Figure 3A, the liver coefficient in the AP group was significantly higher than in the M group (*p* < 0.001). In contrast, in the T, GQ01, and T + GQ01 groups, the liver coefficient decreased and was comparable to that of the NC group. This indicated that the liver weight of HUA mice was greater than that of normal mice, and treatment with allopurinol further enhanced this trend. In the liver, excess uric acid leads to hepatocellular injury through channels such as insulin resistance, oxidative stress, and induction of inflammatory mediator release [25]. AST and ALT are mainly found in the cytoplasm of the liver and are commonly used clinically to evaluate liver function impairment [26]. As shown in Figure 3B,C, compared to group NC, both ALT and AST enzyme activities were increased in group M, with the AST enzyme activity showing a significant increase (*p* < 0.001). This trend was reversed in groups T, T + GQ01, and AP, where the enzyme activities of ALT and AST were significantly lower (*p* < 0.05 and *p* < 0.001). Specifically, in the AP group, ALT activity was notably reduced (*p* < 0.01) and AST activity was significantly diminished (*p* < 0.001). The data indicated that the T + GQ01 group exhibited a stronger synergistic effect compared to the T and GQ01 groups alone, with ALT and AST enzyme activities in the T + GQ01 group being similar to those in the AP group. Integrated with Figure 3D, hepatocytes in the NC group exhibited a rounded, compact structure, featuring large, centrally located nuclei with distinct nucleoli and abundant cytoplasmic content. Conversely, in the M group, manifestations such as the swelling of hepatocytes, mild dilation of hepatic sinusoids, irregular cellular shapes, infiltration of inflammatory cells, and deposition of fat cysts were observed. In the AP group, relative to the M group, hepatic sinusoid dilation was more pronounced, accompanied by reduced hepatocyte volume and nuclear size, along with increased inflammatory cell infiltration (predominantly neutrophilic granulocytes) within the lesions. Besides, the cellular morphology of liver tissue in the T, T + GQ01, and GQ01 groups showed some improvement, especially in the T + GQ01 group, with hepatocyte morphology closely resembling that observed in the NC group. The above results showed that the adverse symptoms of T group, T + GQ01 group, and GQ01 group were significantly improved, indicating that T + GQ01 could alleviate HUA symptoms and improve liver tissue injury.

### 3.4. P. acidilactici GQ01, Buckwheat-Fermented Postbiotics, Collagen Peptide and Multiple Medicinal Food Blends Synergistically Improve the Intestinal Flora Structure in HUA Mice

**Figure 4:**
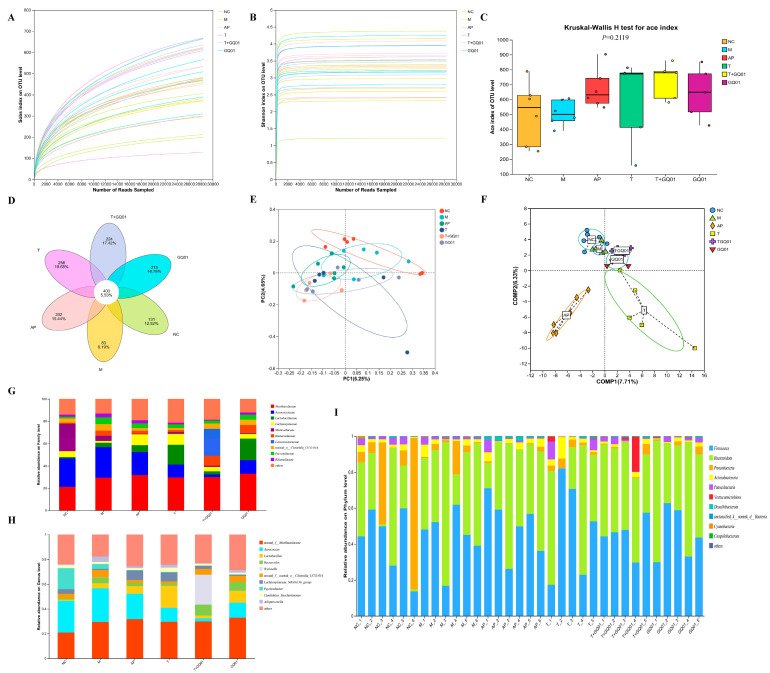
Results of the dilution curves, including (**A**) Sobs index; (**B**) Shannon index; (**C**) Ace index of OTU level; and (**D**) Venn analysis; (**E**) PCoA analysis; (**F**) PLS-DA results at the species level; the community composition at the (**G**) Family, (**H**) Genus, (**I**) Phylum level. Data are presented as mean ± SD (*n* = 6).

Intestinal flora has the role of promoting purine and UA catabolism and increasing uric acid excretion, etc. [10]. Changes or imbalances in the flora structure can cause metabolic disorders, participate in the synthesis of purine-metabolizing enzymes and the release of inflammatory cytokines, which are closely related to the development of HUA [27]. Studies have shown that diets high in purines and fructose can change the structure of the intestinal flora, promote UA production and increase the number of harmful bacteria [28]. The Sobs and Shannon indices flattened once sequences exceeded 30,000 and 5000 (Figure 4A,B). The Kruskal–Wallis H test was performed to analyze the number of OTUs in different groups for the Ace index (Figure 4C). Results showed that there were significant differences in the number of OTUs among different groups (*p* < 0.05). Group T had the highest median value of OTU number, followed by Group T + GQ01, while Group M had the lowest. These findings indicate that the composition of microbial communities, as reflected by the OTU number in relation to the Ace index, varies significantly across different experimental groups. The effects of T, T + GQ01, and GQ01 on the differences in the composition of the intestinal flora of HUA mice are shown in Figure 4D, and the Venn diagrams show that there were a total of 400 OTUs in the six groups of samples, with 131 OTUs specific to the NC group, 83 OTUs specific to the M group, 202 OTUs specific to the AP group, 256 OTUs specific to the T group, 224 OTUs specific to the T + GQ01 group, and 215 GQ01 group-unique OTUs. It indicated the similarity of the intestinal flora of the samples in each group at the OTU level. As can be seen from Figure 4E, the six groups of samples were both close and intersecting as well as separated and analyzed at the OTU level; the NC group was far away from the AP group, indicating that there was a significant difference in the potential principal components of the differences in the composition of the sample communities between the NC group and the AP group, whereas the other groups were close to each other and intersecting, indicating that the sample communities were similar in composition. In the partial least squares discriminant analysis (PLS-DA) at the species level, distinct group-separation patterns were observed (Figure 4F). The score plot of PLS-DA clearly illustrated that the samples from the T group and the T + GQ01 group exhibited an intersecting distribution, indicating a certain degree of similarity in species composition between these two groups. In contrast, both the T and the T + GQ01 group were clearly separated from the AP group, suggesting significant differences in species composition between them. Moreover, the samples of the T + GQ01 group were found to be in close proximity to those of the NC group, demonstrating a relatively high degree of similarity at the species level. At the family level, 10 main types of bacteria were detected in the intestinal flora of the six groups (Figure 4G). The intestinal flora community structure of the samples showed a diversity of 10 species at the genus level (Figure 4H). Analysis at the phylum level revealed differences in the compositional diversity among various groups (Figure 4I). At the genus level, significant shifts in microbial abundance were observed across the experimental groups compared to the NC group. In the M group, the abundance of *Psychrobacter*, *Lachnospiraceae_NK4A136*, and *Candidatus_Saccharimonas* decreased, while the abundance of *Bacteroides*, *Lactobacillus*, *Prevotella*, *norank_f_Muribaculaceae* and *Aerococcus increased*. In the AP group, the abundance of *Psychrobacter* and *norank_f_norank_o_Clostridia_UCG-014* decreased, while *Weissella*, *Lactobacillus*, and *Lachnospiraceae_NK4A136* increased. These results indicated a potential modulation of the intestinal flora towards taxa associated with improved intestinal health, such as *Weissella* and *Lactobacillus*. The T group exhibited an increase in the abundance of *Weissella*, *Lactobacillus*, *Candidatus_Saccharimonas*, and *Lachnospiraceae_NK4A136*, alongside a decrease in *Psychrobacter* and *norank_f_norank_o_Clostridia_UCG-014*. This pattern suggested that the functional complex might enhance the proliferation of beneficial microbes while reducing the presence of less desirable taxa. Within the GQ01 group, a notable increase in the abundance of Psychrobacter, Aerococcus, and Candidatus_Saccharimonas decreased, while *Weissella*, *Lactobacillus*, *Bacteroides*, and *norank_f_norank_o_Clostridia_UCG-014* increased. This highlighted the potential of GQ01 to modulate the intestinal flora by promoting beneficial genera and suppressing others. The T + GQ01 group displayed a distinct microbial community structure compared to all other groups. Notably, the abundance of Weissella and Bacteroides significantly increased, while *Aerococcus* and *Psychrobacter* decreased markedly. This unique profile suggested a synergistic effect of the T and GQ01 interventions, leading to a more pronounced shift towards beneficial microbial taxa. In particular, the T + GQ01 combination demonstrated a unique and potentially beneficial impact on the intestinal flora community, highlighting its potential for further exploration in intestinal health applications.

**Figure 5:**
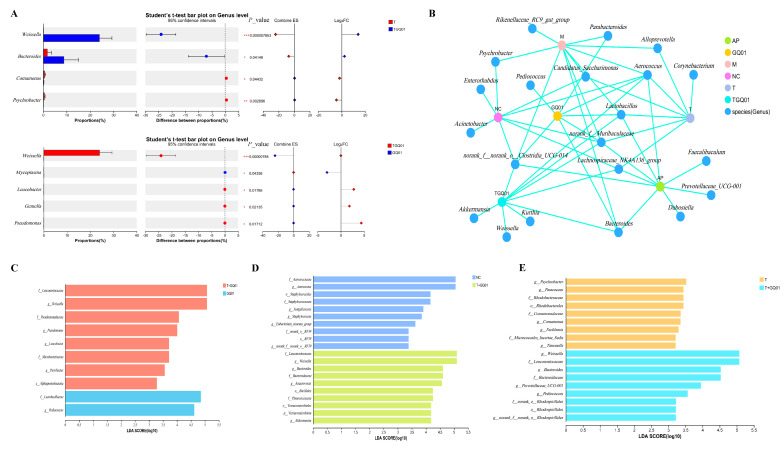
The impact of *P. acidilactici* GQ01 combined with the functional complex composed of buckwheat-fermented postbiotics, collagen peptide, and multiple medicinal food blends on intestinal flora. (**A**) differences in microbial taxa at the genus level among the T group, T + GQ01 group, and GQ01 group; (**B**) correlation between samples and communities as well as between communities (at the genus level); Student’s *t*-test of intestinal flora composition at the genus level, LDA analysis was conducted to compare (**C**) group GQ01 with group T + GQ01; (**D**) group NC with group T + GQ01; and (**E**) group T with group T + GQ01; data are presented as mean ± SD (*n* = 6). *, *p* < 0.05; **, *p* < 0.01; ***, *p* < 0.001.

Species differential analysis uncovered substantial variations in microbial taxa at the genus level across the T, T + GQ01, and GQ01 groups (Figure 5A). Specifically, the abundances of Weissella and Bacteroides in the T + GQ01 group were significantly higher than those in the T group (*p* < 0.05, *p* < 0.01). Additionally, the abundances of *Weissella*, *Leucobacter*, *Gemella*, and *Pseudomonas* in the T + GQ01 group were significantly higher than those in the GQ01 group (*p* < 0.05, *p* < 0.01, *p* < 0.001). In contrast, the abundances of *Comamonas* and *Psychrobacter* in the T + GQ01 group were significantly lower than those in the T group (*p* < 0.05, *p* < 0.01), while the abundance of *Mycoplasma* was significantly lower than that in the GQ01 group (*p* < 0.05, *p* < 0.01). These differences indicated that the introduction of GQ01 significantly altered the microbial community structure, particularly in the T + GQ01 group, where the abundances of *Weissella* and *Bacteroides* were significantly increased. *Weissella*, a type of lactic acid bacteria, is often associated with fermentation processes and probiotic effects [29]. Its increased abundance might suggest that the experimental conditions in the T + GQ01 group were favorable for its growth and metabolic activities. Additionally, *Bacteroides*, a common intestinal commensal, showed increased abundance, possibly reflecting an improved intestinal environment or optimized nutritional conditions in the T + GQ01 group. Notably, the abundances of *Weissella*, *Leucobacter*, *Gemella*, and *Pseudomonas* in the T + GQ01 group were significantly higher than those in the GQ01 group, indicating that the introduction of GQ01 may have promoted the growth of these taxa through certain mechanisms. *Pseudomonas*, a bacterium widely found in the environment, may have increased in abundance due to changes in environmental conditions, while the increased abundances of *Leucobacter* and *Gemella* may reflect shifts in microbial interactions or competitive relationships [30,31,32]. On the other hand, the abundances of *Comamonas* and *Psychrobacter* in the T + GQ01 group were significantly lower than those in the T group, suggesting that the introduction of GQ01 may inhibit the growth of these taxa. *Comamonas*, often associated with organic matter degradation, may be downregulated due to the reduced availability of organic substrates or altered metabolic pathways [33]. *Psychrobacter*, a cold-tolerant bacterium, may be decreased in abundance due to experimental conditions that were unfavorable for its growth [34]. Furthermore, the abundance of Mycoplasma in the T + GQ01 group was significantly lower than that in the GQ01 group, indicating that the introduction of GQ01 may suppress the growth of *Mycoplasma*. As a conditional pathogen, the reduced abundance of *Mycoplasma* may have positive implications for host health [35]. These changes likely reflect the regulatory effects of T + GQ01 on the microbial ecosystem and provide new insights for further research into the interactions between microbial communities and environmental factors. To explore the distribution and correlations between samples and species, as well as among species themselves, we conducted an analysis at the genus level. The results were shown in Figure 5B. The genera *Lactobacillus*, *norank_f_Muribaculaceae*, *norank_k_norank_o_Clostridia_UCG-014*, and *Aerococcus* were found to be correlated with all groups, including the NC, M, AP, T, T + GQ01, and GQ01 group. Bacteroides showed correlations with the M, AP, GQ01 and T + GQ01 groups, while *Lachnospiraceae_NK4A136_group* was correlated with the T, NC, AP, and T + GQ01 groups. Notably, *Akkermansia*, *Weissella*, and *Kurthia* were uniquely correlated with the T + GQ01 group. *Lactobacillus*, a well-known probiotic genus, is often associated with intestinal health and fermentation processes, indicating its potential importance in maintaining microbial balance across various environments [36]. The association of the *Lachnospiraceae_NK4A136_group* with the T, NC, AP, and T + GQ01 groups further underscored the complexity of microbial interactions. Members of the *Lachnospiraceae* family are known for their ability to produce SCFAs, which are essential for intestinal health and metabolic regulation [37]. Their presence in multiple groups indicates their potential role in supporting intestinal integrity and metabolic functions [38]. The unique correlation of *Akkermansia*, *Weissella*, and *Kurthia* with the T + GQ01 group suggested that the introduction of GQ01 might have created a unique ecological niche favoring these genera. *Akkermansia*, particularly *Akkermansia muciniphila*, is renowned for its beneficial effects on intestinal barrier function and metabolic health [39]. Its presence in the T + GQ01 group might indicate improved intestinal health and metabolic regulation due to GQ01 intervention. *Weissella*, a genus of lactic acid bacteria, is often associated with probiotic properties and fermentation processes, suggesting that its enrichment in the T + GQ01 group might contribute to enhanced microbial stability and functionality. *Kurthia*, although less studied in the context of intestinal flora, might play a role in niche-specific interactions within the T + GQ01 group. Based on the Linear Discriminant Analysis Effect Size (LEfSe) multi-level species difference discrimination analysis (from phylum to genus), difference tests were carried out at multiple hierarchical levels. Differentially abundant species at multiple levels were analyzed, and the Linear Discriminant Analysis (LDA) score was used to measure the impact of species on the differential effect. These findings indicated that these species might play a crucial role in the process of environmental change. The results respectively compared the differentially abundant species between the T + GQ01 group and the GQ01 group, the NC group and the T group (Figure 5C–E). The comparative analysis of microbial communities revealed significant shifts in the intestinal microbiota composition of the T + GQ01 group relative to the control groups. Compared to the GQ01 group, the T + GQ01 group exhibited a marked increase in the relative abundance of *Leuconostocaceae*, *Weissella*, *Pseudomonas*, *Leucobacter*, *Microbacteriaceae*, *Parvibacter*, and *Alphaproteobacteria*. Notably, *Weissella* and *Leuconostocaceae* are well-documented for their roles in lactic acid production and their potential probiotic effects, which may contribute to intestinal health by enhancing barrier function and modulating immune responses [40]. The enrichment of *Pseudomonas* and *Alphaproteobacteria* suggests potential metabolic adaptations, as these taxa are often associated with diverse metabolic capabilities, including the degradation of complex carbohydrates [41,42]. When compared to the NC group, the T + GQ01 group showed a significant increase in *Leuconostocaceae*, *Weissella*, *Bacteroides*, *Bacteroidaceae*, *Anaeroidaceae*, *Anaerovorax*, *Bacillales*, *Planococcaceae*, *Verrucomicrobiales*, *Verrucomicrobiota*, and *Akkermansia*. The elevated levels of *Bacteroides* and *Akkermansia* are particularly noteworthy, as these taxa are strongly associated with improved intestinal barrier integrity and anti-inflammatory effects [43]. *Akkermansia* in particular has been widely recognized for its role in mucin degradation and its potential to mitigate metabolic disorders [44]. The presence of *Verrucomicrobia* further supports the potential for enhanced mucosal health, as this phylum is often linked to a healthy intestinal environment [45]. Relative to the T group, the T + GQ01 group demonstrated a higher abundance of *Leuconostocaceae*, *Bacteroides*, *Prevotellaceae_UCG-001*, *Pediococcus*, and *Rhodospirillales*. The increased prevalence of *Prevotellaceae_UCG-001* and *Rhodospirillales* suggests a potential shift toward microbial communities capable of fermenting dietary fibers and producing SCFAs, which are critical for maintaining intestinal homeostasis and systemic health [46]. These findings aligned with previous studies demonstrating that dietary interventions and microbial supplementation can selectively enrich beneficial taxa, thereby modulating intestinal flora composition and function [47,48]. The observed shifts in microbial populations highlighted the synergistic effects of the T + GQ01 intervention, which might promote intestinal health through the enrichment of functionally significant taxa.

### 3.5. P. acidilactici GQ01, Buckwheat-Fermented Postbiotics, Collagen Peptide, and Multiple Medicinal Food Blends Can Synergistically Improve Intestinal SCFAs Content

SCFAs are the main metabolites of intestinal flora. Studies have confirmed that acetic acid helps to enhance host immunity [49]. However, recent findings suggested that SCFAs, such as acetic acid, are beneficial to host health but can be harmful to the host at too high a level [50]. Propionic acid inhibits intracellular lipid degradation and improves the lipid buffering capacity of adipose tissues, and butyric acid helps to maintain the integrity of the intestinal barrier [51]. To explore the impacts of T, T + GQ01, and GQ01 on the alterations of intestinal flora in HUA, the SCFAs content in the cecal contents of each group was compared, and the results are presented in Figure 5. In comparison with the NC group, the total SCFA content in the M group was relatively increased. In the AP group, the acetic acid content decreased, while the contents of butyric, isobutyric and isovaleric acids increased. Moreover, the contents of propionic acid and valeric acid were significantly elevated (*p* < 0.01). Group T exhibited significantly lower acetic acid content (*p* < 0.001) but higher levels of propionic, butyric, isobutyric, valeric, and isovaleric acids (*p* < 0.01). Group GQ01 showed a similar trend, with significantly reduced acetic acid (*p* < 0.001), and increased propionic, butyric, valeric, isobutyric, and isovaleric acids (*p* < 0.001). In group T + GQ01, acetic acid content was significantly lower (*p* < 0.001), while propionic, butyric, valeric, isobutyric, and isovaleric acids were significantly elevated (*p* < 0.001). Compared to group M, total SCFAs were elevated in group AP, with significant increases in acetic, propionic, valeric, isobutyric, and isovaleric acids (*p* < 0.001). Group T also demonstrated significantly higher levels of these acids (*p* < 0.001), with a notable increase in butyric acid. In the T + GQ01 group, acetic acid content was significantly reduced (*p* < 0.001), while propionic, butyric, valeric, and isovaleric acids were significantly increased (*p* < 0.001); isobutyric acid content showed an upward trend but without statistical significance. Similarly, in the GQ01 group, acetic acid content was significantly lower (*p* < 0.001), and propionic, butyric, valeric, and isovaleric acids were significantly higher (*p* < 0.01), with isobutyric acid content also trending upward but not significantly. These results indicated that T, T + GQ01, and GQ01 improved SCFA abundance in HUA mice, suggesting that T, T + GQ01, and GQ01 have the effect of protecting the intestinal barrier, improving intestinal function and maintaining intestinal health. The changes in SCFA levels, particularly the increase in propionic and butyric acids, indicate improved intestinal barrier function and metabolic health. These findings suggested that T, T + GQ01, and GQ01 interventions could effectively restore intestinal flora balance and enhance SCFA production, offering potential therapeutic benefits for HUA and related metabolic disorders. The elevated SCFAs, especially butyrate and propionate, likely mediate these protective effects through multiple mechanisms including strengthening intestinal tight junctions (ZO-1, occludin) [52], providing energy for colonocytes, exerting anti-inflammatory effects via HDAC inhibition, modulating systemic immunity and insulin sensitivity through GPR43/41 signaling [53], and potentially alleviating HUA by regulating renal urate transporters (ABCG2/URAT1) [54,55]. These mechanistic insights, supported by recent studies, further validate the therapeutic potential of targeting the gut microbiota–SCFA axis for HUA management.

### 3.6. Effect of P. acidilactici GQ01 Combined with Buckwheat-Fermented Postbiotics, Collagen Peptide and Multiple Medicinal Food Blends on Spearman Correlation Analysis of Intestinal Flora in HUA Mice

**Figure 6:**
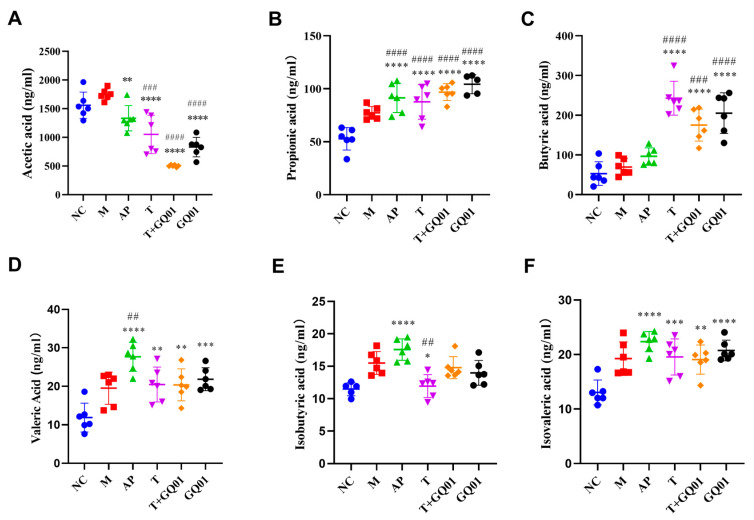
Effects of T, T + GQ01, and GQ01 on the levels of SCFAs in colonic metabolites of HUA model mice. (**A**) Acetic acid; (**B**) Propionic acid; (**C**) Butyric acid; (**D**) Valeric acid; (**E**) Isobutyric acid; and (**F**) Isovaleric acid content. Data are presented as mean ± SD (*n* = 6).* *p* < 0.05, ** or ^##^ *p* < 0.01, *** or ^###^ *p* < 0.001, **** or ^####^ *p* < 0.0001 (^#^ Contrasted with the NC group and * was contrasted with M Group).

Through Spearman correlation analysis, the microbiota significantly associated (either positively or negatively) with HUA can be identified. These microbiota could serve as potential biomarkers or therapeutic targets [28]. The results are shown in Figure 6. Serum UA levels were significantly negatively correlated with the abundance of *norank_f_Desulfovibrionaceae*, *norank_f_Oscillospiraceae*, *Desulfovibrio*, and *norank_f_Ruminococcaceae*, suggesting that these microbial taxa may play a role in reducing UA levels. In contrast, serum UA levels were positively correlated with *acinetobacter*, indicating that *acinetobacter* might be associated with promoting UA production or inhibiting UA excretion [56]. The microbial taxa influencing changes in CR and BUN were similar. Both CR and BUN levels were positively correlated with *norank_f_Desulfovibrionaceae* and *norank_f_norank_o_Clostridia_vadinBB60_group*, suggesting that these taxa might influence the nitrogen metabolism or kidney excretion, but negatively correlated with *Comamonas* and *Weissella*, implying potential protective roles of these taxa in kidney function [47]. Additionally, BUN levels were positively correlated with *Faecalibaculum*, *Dubosiella*, *Alistipes*, and *Lachnospiraceae_NK4A136_group*. The microbial taxa associated with changes in ALT and AST levels differed. ALT levels were negatively correlated with *norank_f_Desulfovibrionaceae* and *Pediococcus*, but positively correlated with *Candidatus_Saccharimonas*, *Psychrobacter*, and *norank_f_norank_o_RF39*. AST levels were also associated with *norank_f_Oscillospiraceae*, *Aerococcus*, and *unclassified_f_Prevotellaceae*, further supporting the complex interplay between intestinal flora and liver health. The enzymatic activity of ADA and XOD also showed significant correlations with specific microbiota. ADA activity was negatively correlated with *norank_f_norank_o_RF39*, suggesting a potential regulatory role of this taxon in adenosine metabolism. Conversely, the positive correlations of ADA activity with *Comamonas*, *Kurthia*, and *Desulfovibrio* highlight their possible involvement in purine catabolism. The negative correlation between XOD activity and *norank_f_Desulfovibrionaceae* and *Faecalibaculum* implies their potential to suppress UA production, while the positive correlation with *Acinetobacter* and *Comamonas* aligns with their pro-uricemic effects. In conclusion, this study demonstrated that specific intestinal flora might be closely associated with serum UA levels, kidney function markers (CR and BUN), liver enzymes (ALT and AST), and key enzymatic activities (ADA and XOD). These findings provided new insights into the intestinal flora–host metabolic axis and suggested potential microbial targets for managing HUA, kidney dysfunction, and liver-related disorders.

**Figure 7:**
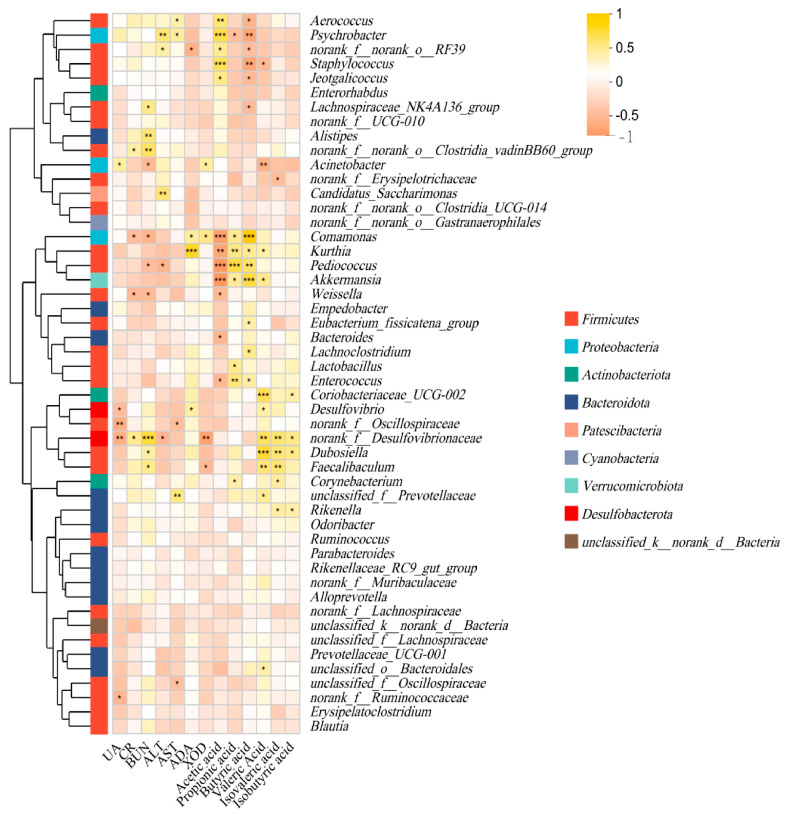
Analysis of the correlation between intestinal flora and SCFAs and Serum biochemistry. Data are presented as mean ± SD; * *p* < 0.05, ** *p* < 0.01, and *** *p* < 0.001.

The intestinal flora plays a pivotal role in maintaining host health and intestinal homeostasis, largely through the production of SCFAs, which serve as key signaling molecules and energy sources [57]. Spearman correlation analysis revealed significant correlations between specific SCFAs and intestinal flora taxa, providing insights into the complex interactions between microbiota and host metabolism (Figure 7). Acetate levels were negatively correlated with *Akkermansia*, *Comamonas*, *Enterococcus*, *Kurthia*, *Weissella*, and *Bacteroides*, but positively correlated with *Aerococcus*, *Psychrobacter*, *Staphylococcus*, *norank_f_norank_o_RF39*, and *Jeotgalicoccus*. Valerate levels were positively correlated with *Desulfovibrio*, *unclassified_o_Bacteroidales*, *unclassified_o_Prevotellaceae*, *Coriobacteriaceae_UCG-002*, *Dubosiella*, *norank_f_Desulfovibrionaceae*, *Faecalibaculum*, *Kurthia*, and *Akkermansia*, but negatively correlated with *Staphylococcus* and *Acinetobacter*. Isovalerate levels were positively correlated with *Faecalibaculum*, *norank_f_Desulfovibrionaceae*, and *Dubosiella*, but negatively correlated with *norank_f_Erysipelotrichaceae*. Isobutyrate levels were positively correlated with *norank_f_Desulfovibrionaceae*, *Dubosiella*, *Rikenella*, and *Coriobacteriaceae_UCG-002*. Propionate levels were positively correlated with *Akkermansia*, *Comamonas*, *Enterococcus*, *Kurthia*, *Lactobacillus*, and *Corynebacterium*, but negatively correlated with *Psychrobacter*. Butyrate levels were positively correlated with *Akkermansia*, *Comamonas*, *Enterococcus*, *Kurthia*, *Eubacterium_fissicatena_group*, and *Lachnoclostridium*, but negatively correlated with *Lachnospiraceae_NK4A136_group*, *Aerococcus*, *Psychrobacter*, *Staphylococcus*, *norank_f_norank_o_RF39*, and *Jeotgalicoccus*. The observed correlations between SCFAs and specific microbial taxa underscore the functional diversity of the intestinal flora in SCFA metabolism. These findings suggested that manipulating the abundance of key microbial taxa, such as *Akkermansia*, *Desulfovibrio*, and *Lactobacillus* could modulate SCFA level host health consequently.

## 4. Conclusions

This study elucidated the synergistic effects of *P. acidilactici* GQ01 and a functional complex composed of buckwheat-fermented postbiotics, collagen peptide and multiple medicinal food blends (T) in ameliorating HUA through the modulation of intestinal flora. The intervention demonstrated significant regulatory effects on intestinal flora composition and function, which in turn influenced systemic uric acid metabolism, renal excretion, and hepatic uric acid production. By targeting the gut–kidney axis, the treatment enhanced renal urate excretion and reduced renal inflammation, while modulation of the gut–liver axis contributed to the suppression of hepatic xanthine oxidase activity. The combination therapy (T + GQ01) demonstrated superior efficacy compared to individual treatments, underscoring its potential as a novel therapeutic approach. T + GQ01 significantly reduced serum UA levels by enhancing ADA activity and inhibiting XOD, key enzymes in purine metabolism. This dual action not only lowered UA levels but also mitigated the adverse effects of HUA on kidney and hepatic functions.

In the kidney metabolic system, T + GQ01 alleviated kidney injury by reducing serum CRE and BUN levels and improving histopathological features such as glomerular atrophy and tubular dilation. The hepatic benefits were evident through decreased ALT and AST activities, indicating improved liver function. Histological analysis further confirmed the protective effects of T + GQ01 on liver tissue, reducing inflammation and cellular damage.

Moreover, T + GQ01 modulated the intestinal flora, promoting the growth of beneficial genera like *Weissella* and *Bacteroides*, while suppressing harmful taxa such as *Psychrobacter* and *Acinetobacter*. This modulation led to increased production of SCFAs, particularly propionic and butyric acids, which are crucial for maintaining intestinal barrier integrity and overall metabolic health. The unique microbial community structure in the T + GQ01 group, characterized by higher abundances of *Weissella* and *Bacteroides*, suggests a synergistic effect that enhances intestinal health and systemic metabolism. This study systematically investigated the relationship between gut microbial communities and SCFA production, demonstrating the complex functional interactions between microbial ecosystem composition and host metabolic outcomes. These findings contributed to our understanding of the intestinal flora–host metabolic axis and offer potential targets for dietary or probiotic interventions aimed at optimizing intestinal health and preventing metabolic disorders.

In conclusion, the combination of *P. acidilactici* GQ01, buckwheat-fermented postbiotics, collagen peptide and multiple medicinal food blends presents a cocktail-like regulatory approach for the management of HUA. This approach targeted multiple aspects, including various stages of purine metabolism, kidney, and hepatic functions, along with the structural and functional dynamics of intestinal flora ecosystems. These findings underscored the importance of integrating probiotic, prebiotic, and postbiotic interventions in the treatment of metabolic disorders.

## Data Availability

The original contributions presented in the study are included in the article; further inquiries can be directed to the corresponding author.
